# A Novel Angiogenesis-Related Gene Signature to Predict Biochemical Recurrence of Patients with Prostate Cancer following Radical Therapy

**DOI:** 10.1155/2022/2448428

**Published:** 2022-06-28

**Authors:** Bohan Fan, Yicun Wang, Xin Zheng, Xin Zhang, Zijian Zhang, Xiaopeng Hu

**Affiliations:** ^1^Department of Urology, Beijing Chao-Yang Hospital, Capital Medical University, Beijing, China; ^2^Institute of Urology, Capital Medical University, Beijing, China; ^3^Comprehensive Transplant Center, Northwestern University Feinberg School of Medicine, Chicago, IL, USA; ^4^Department of Surgery, Northwestern University Feinberg School of Medicine, Chicago, IL, USA; ^5^Department of Urology, Beijing Youan Hospital, Capital Medical University, Beijing, China

## Abstract

**Background:**

Prostate cancer (PCa) is one of the most common malignancies in males; we aim to establish a novel angiogenesis-related gene signature for biochemical recurrence (BCR) prediction in PCa patients following radical therapy.

**Methods:**

Gene expression profiles and corresponding clinicopathological data were acquired from The Cancer Genome Atlas (TCGA) and Gene Expression Omnibus (GEO) database. We quantified the levels of various cancer hallmarks and identified angiogenesis as the primary risk factor for BCR. Then machine learning methods combined with Cox regression analysis were used to screen prognostic genes and construct an angiogenesis-related gene signature, which was further verified in external cohorts. Furthermore, estimation of immune cell abundance and prediction of drug responses were also conducted to detect potential mechanisms.

**Results:**

Angiogenesis was regarded as the leading risk factor for BCR in PCa patients (HR = 1.58, 95% CI: 1.38–1.81), and a novel prognostic signature based on three genes (*NRP1*, *JAG2*, and *VCAN*) was developed in the training cohort and successfully validated in another three independent cohorts. In all datasets, this signature was identified as a prognostic factor with promising and robust predictive values. Moreover, it also predicted higher infiltration of regulatory *T* cells and *M*2-polarized macrophages and increased drug sensitivity of angiogenesis inhibitors in high-risk patients.

**Conclusions:**

The angiogenesis-related three-gene signature may serve as an independent prognostic factor for BCR, which would contribute to risk stratification and personalized management of PCa patients after radical therapy in clinical practice.

## 1. Introduction

As one of the most prevalent malignancies in aging males, prostate cancer (PCa) ranks second in terms of mortality rate according to the latest cancer statistics [1]. Most patients with localized cancer receive standard therapy such as radical prostatectomy (RP) or radical radiotherapy (RT) [2]. However, approximately 20%–40% of patients with RP and 30%–50% of patients with RT will develop biochemical recurrence (BCR) within ten years [3], which is defined as consecutive rising prostate-specific antigen (PSA) values above 0.2 ng/ml twice following RP or >2 ng/ml higher than the nadir PSA values following RT [4]. PCa patients with BCR showed a higher probability of clinical recurrence and underlying metastasis, and thus, the early recognition of BCR is of crucial importance for subsequent treatments [5]. Currently, clinicopathological parameters, consisting of Gleason score, TNM stage, and PSA have been introduced but are insufficient to predict BCR [6], and more accurate methods are urgently needed to better stratify and earlier identify BCR of PCa patients after radical therapy.

Angiogenesis, a dynamic process that involves interactions between endothelial cells and the extracellular environment, has been proved to play a vital role in the spread and development of PCa [7, 8]. Besides, higher microvessel density indicates progression, metastasis, and worse prognosis [9], and therefore, targeting angiogenesis has been the aim of several clinical investigations and would be a promising treatment strategy for PCa [10, 11]. Moreover, measurement of angiogenetic activity holds great potential in prognostic prediction and markers used to assess antiangiogenic treatment response would be beneficial to patient management in clinical practice [11].

In recent years, molecular markers show outstanding interpretability and predictive power in improving the diagnosis, prognosis, and therapy of urological malignancies [12]. Particularly, previous studies focused on gene expression data to develop signatures in discriminating BCR [13, 14]. However, similar limitations exist in most research. For example, there are too many genes included in several signatures, which are technically difficult and too expensive to perform in clinical settings [5]. Besides, signatures were independently verified in few validation cohorts and may be limited by the lack of wide application. In the current research, we firstly explored the association between cancer hallmarks and BCR, thus identifying the angiogenesis-associated activities as the primary risk factors leading to recurrence in PCa patients after radical therapy. Subsequently, machine learning methods combined with Cox regression analysis were performed to construct an angiogenesis-related signature to predict BCR, which was further verified in another three independent cohorts. Furthermore, drug sensitivity prediction, analyses of functional enrichment, and immune cell infiltration would also provide novel insights into the mechanisms of PCa.

## 2. Methods

### 2.1. Data Collection and Preprocessing

A total of 844 patients included in four datasets were enrolled in our research based on the following criteria: (1) patients with primary PCa followed radical radiotherapy or prostatectomy; (2) patients with gene expression profiles of tumor biopsies and corresponding clinical information (i.e., BCR event, time to BCR, Gleason score, and total follow-up time); (3) patients with follow-up time more than 30 days; and (4) datasets with over 50 eligible samples. The RNA-Seq data of 327 PCa patients was assessed from The Cancer Genome Atlas (TCGA) and was utilized as the training cohort to construct an angiogenesis-related gene signature. In addition, the microarray data of 223 patients from GSE116918 (Almac Diagnostics Prostate Disease Specific Array (DSA)) [15] and 221 patients from GSE70770 (Illumina HumanHT-12 V4.0 expression beadchip) [16] and the RNA-seq data of 93 patients from GSE54460 (Illumina HiSeq 2000) were downloaded from Gene Expression Omnibus (GEO) database [17], all of them were separately used as independent validation cohorts. Main characteristics of the above datasets can be seen in Table 1. All RNA-seq and microarray data included in this study were normalized and log_2_-transformed.

### 2.2. Study Design

**Figure 1:**
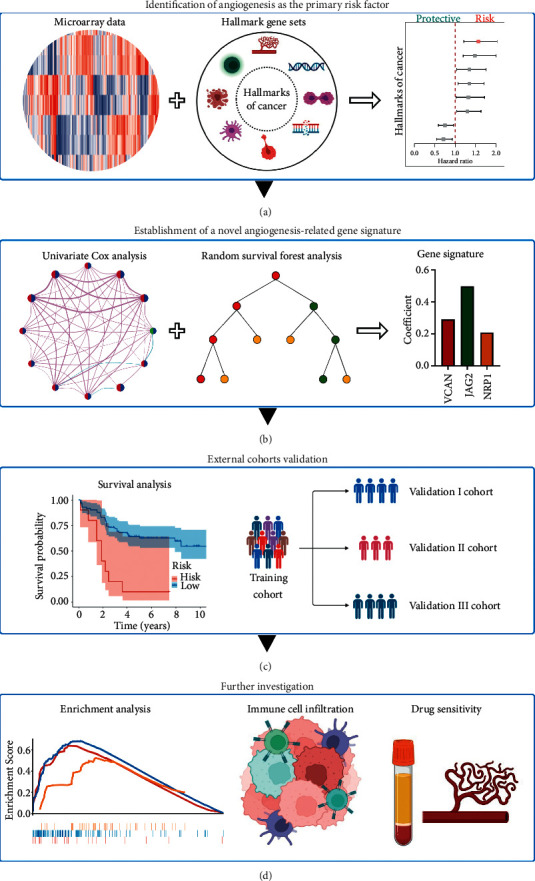
Flowchart of this study. Four stages are included in our study. Firstly, angiogenesis was identified as the primary risk factor for BCR by single sample gene set enrichment analysis and survival analysis. Secondly, random survival forest and cox regression analyses screened prognostic genes and constructed an angiogenesis-related gene signature. Thirdly, the predictive ability of the novel signature was further verified in another three validation cohorts. Moreover, functional enrichment and immune cell infiltration analyses and drug sensitivity prediction were also conducted to perform further investigation.

As illustrated in Figure 1, four stages were included in this research. By applying single sample gene set enrichment analysis (ssGSEA), the activities of cancer hallmarks were initially quantified and then univariate Cox analysis identified angiogenesis as the primary risk factor for BCR. After random survival forest analysis, angiogenesis-related prognostic genes were applied to multivariate Cox analysis to construct a gene signature, and the signature was further verified among independent validation cohorts. In the phases of further investigation, we performed enrichment analysis, immune cell infiltration estimation, and drug sensitivity prediction to prove its reliability from a functional perspective.

### 2.3. Hallmark Selection in BCR

In the training cohort, the performances of cancer hallmarks were quantified by ssGSEA algorithm (“GSVA” *R* package) based on transcriptional profiles and gene signatures derived from the Molecular Signatures Database (MSigDB) [18, 19]. Subsequently, we employed univariate Cox analysis to evaluate the significance of various cancer hallmarks in BCR of PCa patients through “survival” *R* package. Angiogenesis with the highest hazard ratios (HRs) was included for further analysis, and based on the angiogenesis-related score, patients were then divided into high- and low-score groups. Subsequently, the survival differences, recurrence rate, immune, and stromal scores (“estimate” *R* package) were evaluated and compared between groups [20].

### 2.4. Construction of the Angiogenesis-Related Gene Signature

Thirty-two genes involved in the processes of angiogenesis were acquired from MSigDB to perform further analyses. Then we applied two approaches to select potential angiogenesis-related genes with prognostic values. Univariate Cox analysis was first used to identify prognostic genes with the threshold of *P* < 0.05. Next, random survival forest (RFS) analysis, an ensemble-tree-based method that adapts random forests to survival analysis, was used to select genes by the minimal depth and variable importance (VIMP) [21]. The minimal depth suggests the average depth of genes among all survival trees and smaller values indicate greater importance, while VIMP measures changes in the predictive ability of the RSF model when variables are randomly permuted, higher scores imply increased importance. Since both of them evaluate the impact of variables from different perspectives, genes commonly selected by minimal depth, and VIMP were included for signature construction. Intersected genes recognized by univariate Cox and RFS analyses were then employed to multivariate Cox analysis to construct a gene signature, the angiogenesis-related score (ARS) was calculated as the expression levels multiplied its corresponding regression coefficient.

### 2.5. Independence and External Validation of ARS

To determine whether the ARS was independent of traditional clinical features (i.e., age, Gleason score, and pathologic *T* stage) in BCR prediction, univariate Cox analysis was performed in all of the training and validation cohorts. The predictive performances of ARS were also measured by employing time-dependent receiver operating characteristic (ROC) curves through “timeROC” *R* package. Besides, PCa patients were divided into high- and low-risk groups through *X*-tile software based on individual ARS, and survival differences were compared in all cohorts.

### 2.6. Gene Set Enrichment Analysis

Before analysis, a GEO-meta cohort was created as the whole validation cohort for further investigation by merging three GEO datasets through “SVA” *R* package. Subsequently, we performed gene set enrichment analysis (GSEA) to compare the enriched pathways between high- and low-risk groups, and the GO, KEGG, hallmark gene sets taken from MSigDB were used as a reference [19].

### 2.7. Immune Cell Infiltration Estimation by Deconvolution Algorithm

CIBERSORT (Cell-type Identification by Estimating Relative Subsets of RNA Transcripts) is a deconvolution algorithm using gene expression profiles to characterize immune cell composition [22]. In this way, the abundance of 22 immune cell subpopulations in the tumor microenvironment of PCa biopsies was evaluated and compared between high- and low-risk groups.

### 2.8. Prediction of Antiangiogenic Therapy Response

Based on “oncoPredict” *R* package, a useful tool to estimate the half-maximal inhibitory concentration (IC_50_) thus predicting therapeutic responses for each sample through ridge regression, antiangiogenic responses of PCa patients to three angiogenesis inhibitors (cabozantinib, lenalidomide, and cediranib) were individually predicted [23]. Genomic expression profiles of considerable cell lines and corresponding drug response data measured with IC_50_ in the Genomics of Drug Sensitivity in Cancer (GDSC) database were utilized as references [24].

## 3. Results

### 3.1. Angiogenesis Was the Primary Risk Factor for BCR in PCa

**Figure 2:**
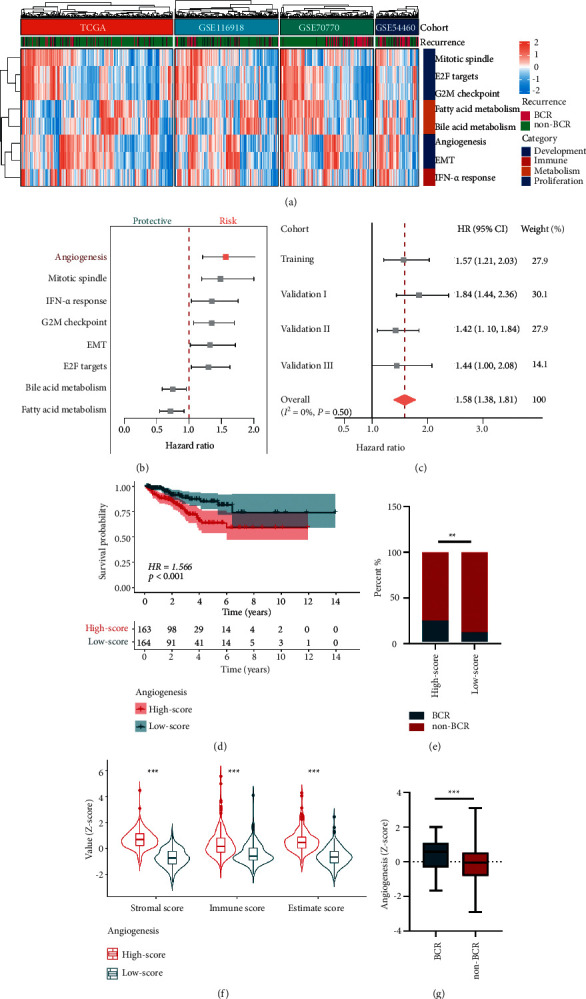
Identification of angiogenesis as the leading risk factor in BCR of PCa patients. (a) Heatmap shows the ssGSEA score of prognostic hallmark activities in four datasets. Angiogenesis ranks first among prognostic factors leading to BCR (b) and its influence on BCR was further validated through meta-analysis (c). (d) Kaplan–Meier (K-M) survival curves of BCR survival between high- and low-score groups stratified by the angiogenesis score. (e) Percentile chart of BCR distribution between high- and low-score groups. (f) Violin plot illustrated different stromal, immune, and estimate scores between groups. (g) Box plot of normalized angiogenesis-related score in BCR or non-BCR patients. BCR: biochemical recurrence. ^*∗∗*^*P* < 0.01; ^*∗∗∗*^*P* < 0.001.

In the training cohort, based on recurrence survival information and ssGSEA scores of cancer hallmarks, the HR value of each hallmark was calculated and ranked (Supplementary Table S1). Intriguingly, cancer hallmarks belonging to development, immune, metabolism, and proliferation categories were significantly associated with BCR (Figures 2(a) and 2(b)). In comparison with other hallmarks, such as IFN-*α* response and epithelial-mesenchymal transition (EMT), angiogenesis exhibited the most powerful effect on recurrence survival (Figure 2(b)). Similar results were also obtained in other validation cohorts, and meta-analysis illustrated a pooled HR of 1.58 for the angiogenesis hallmark (Figure 2(c)). In the training cohort, 327 patients were categorized into high- and low-score groups according to the median angiogenesis-related ssGSEA score, and the high-score group exhibited worse recurrence survival with HR = 1.566 and *P* < 0.001 (Figure 2(d)). Besides, more BCR occurred in patients of the high-score group and the *Z*-scores of angiogenesis were significantly elevated in patients with BCR during follow-up (Figures 2(e) and 2(g)), and higher estimate, immune, and stromal scores were also seen in the high-score group (Figure 2(f)).

### 3.2. Establishment of an Angiogenesis-Related Gene Signature for BCR

**Figure 3:**
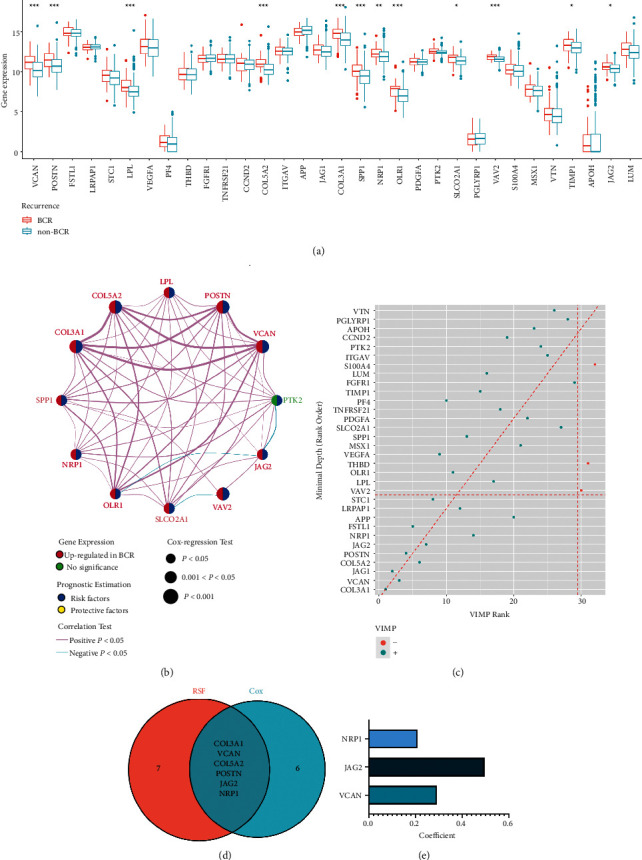
Construction of the angiogenesis-related gene signature. (a) Box plot shows the aberrant expression levels of angiogenesis-related genes in BCR in comparison with non-BCR patients. (b) The interaction of 12 angiogenesis-related prognostic genes in PCa. Expression differences between BCR and non-BCR tissues were depicted in different colors. Genes upregulated in BCR: red; genes with no significant alterations: green. Risk factors are colored in blue while protective factors are depicted in yellow. The lines connecting genes represented their interaction with each other. The size of each circle represents the prognosis effect of each gene scaled by *P* value. (c) Variable importance plot of the random survival forest analysis comparing rankings between minimal depth and variable of importance (VIMP). The VIMP rank is reported on the *x*-axis. The minimal depth (rank order) is on the *y*-axis. The vertical line divides variables with positive VIMP (left) from those with negative VIMP (right; unimportant). The horizontal line indicates the minimal depth threshold: important variables are below the line. The variables on the diagonal red line are those ranked equally by the two methods. (d) Venn diagram shows commonly selected six genes by univariate cox regression and random survival forest analyses. (e) Bar graph displays coefficients of each gene included in the signature.

Among thirty-two angiogenesis-related genes, the expression levels of almost all genes were upregulated in biopsies of PCa patients who underwent BCR (Figure 3(a)), while 12 prognostic genes were all risk factors and they exhibited tight associations with each other (Figure 3(b)). In the following RSF analysis, eleven genes selected by both minimal depth and VIMP were included for subsequent analyses (Figure 3(c)). Through univariate Cox and RSF analyses, six intersected genes (*COL3A1*, *VCAN*, *COL5A2*, *POSTN*, *JAG2*, and *NRP1*) were eligible for multivariate Cox regression analysis (Figure 3(d)). As a result, an angiogenesis-related gene signature involving only three genes (*NRP1*, *JAG2*, and *VCAN*) was constructed (Figure 3(e)). ARS was established by the gene signature and calculated as follows: ARS = (0.290715 *∗* expression level of *VCAN*) + (0.495663 *∗* expression level of *JAG2*) + (0.208562 *∗* expression level of *NRP1*). The expression levels of each gene were normalized and log_2_-transformed.

### 3.3. ARS Served as an Independent Prognostic Factor for BCR in Each Cohort

**Figure 4:**
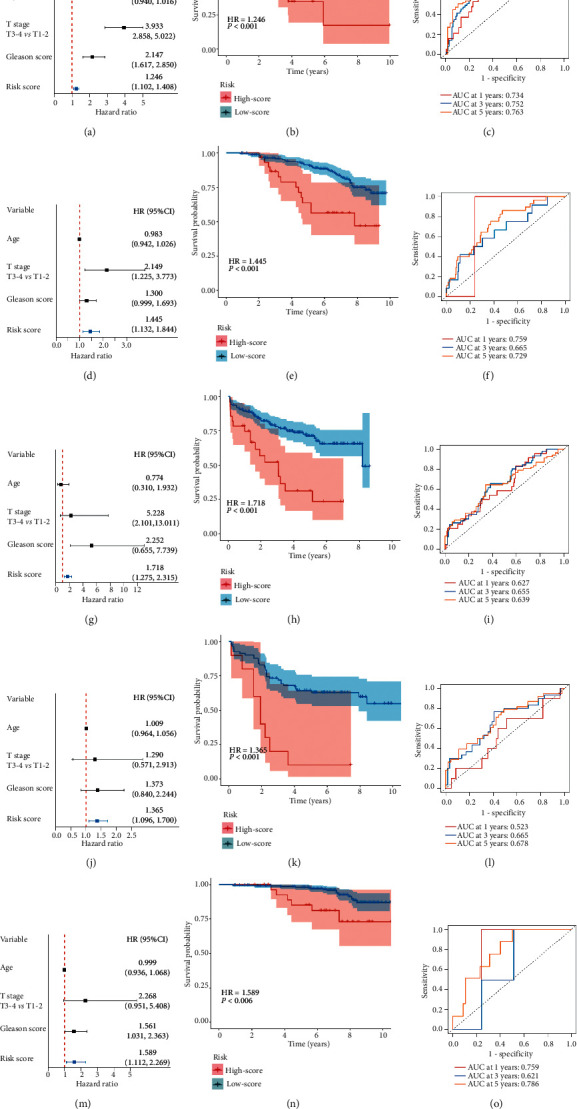
Angiogenesis-related gene signature serves as an independent prognostic factor with promising and robust predictive values. Forest plot illustrates the risk score calculated by angiogenesis-related gene signature is an independent risk factor for BCR in the training (a), validation I (d), validation II (g), and validation III (j) cohorts, respectively. K-M survival curves show poor prognosis in high-risk patients divided by the gene signature in four cohorts (b), (e), (h), and (k). ROC curves illustrate the promising and stable predictive ability of the gene signature in four cohorts (c), (f), (i), and (l). This novel signature can also be a prognostic factor for metastasis (m), and it is suitable for risk stratification and metastasis prediction in the validation I cohort (n), (o).

To verify the independency of ARS in recurrence survival prediction, univariate Cox analysis was performed for ARS and other three clinicopathological factors (age, pathologic *T* stage, and Gleason score), results illustrated that ARS was consistently a risk factor for BCR in all cohorts (Figures 4(a), 4(d), 4(g), and 4(j)). Besides, the Kaplan–Meier (K-M) survival analysis also indicated that high-risk group had significantly unfavorable prognoses than low-risk group (Figures 4(b), 4(e), 4(h), and 4(k)), while the time-dependent ROC curves demonstrated that ARS had reliable predictive abilities across cohorts (Figures 4(c), 4(f), 4(i), and 4(l)). Clinically, early BCR was an essential indicator for distant metastasis of PCa; therefore, the predictive ability of ARS was also evaluated in the Validation I cohort with metastasis-survival information. Results showed that ARS was a primary risk factor with promising performance in prediction for metastasis of PCa (Figures 4(m), 4(n), and 4(o)).

### 3.4. Gene Set Enrichment Analysis

**Figure 5:**
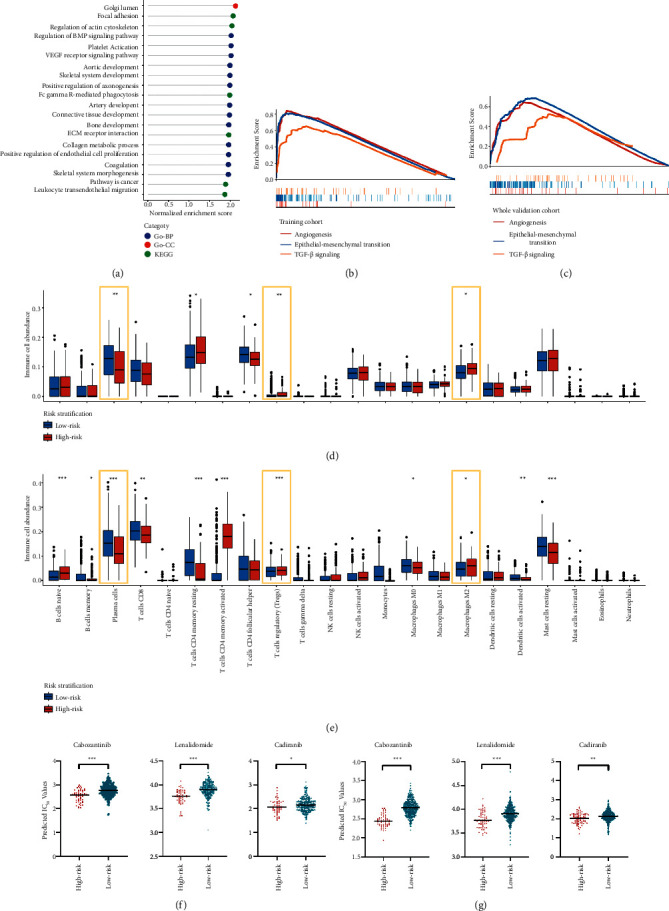
Functional enrichment analysis, immune cell estimation, and drug sensitivity prediction in high- and low-risk groups. Lollipop plot shows pathways significantly enriched in high-risk groups in both training and whole validation cohorts (a). GSEA plots of enriched hallmark activities in the training (b) and whole validation cohorts (c). Box plots show the abundance of immune cells between high- and low-risk groups in the training (d) and whole validation cohorts (e). Bee graph of the predictive IC_50_ values for three anti-angiogenesis agents in the training (f) and whole validation cohorts (g). IC_50_: half-maximal inhibitory concentration. ^*∗*^*P* < 0.05; ^*∗∗*^*P* < 0.01; ^*∗∗∗*^*P* < 0.001.

Gene sets in GO and KEGG were utilized to identify pathways or processes that were differentially enriched in high- and low-risk groups. Results illustrated that angiogenesis-related processes including “platelet activation,” “VEGF receptor signaling pathway,” and “aortic development” were enriched in the high-risk group of both training and whole validation cohorts (Figure 5(a)), indicating that the novel gene signature was of angiogenesis-related characteristics. Besides, cancer hallmarks consisting of “epithelial-mesenchymal transition” and “TGF-*β* signaling” were also significantly enriched in the high-risk group (Figures 5(b) and 5(c)), further confirming that ARS would be an effective tool in screening patients with poor prognosis from the functional perspective.

### 3.5. Inference of Immune Cell Infiltration

The abundance of 22 subtypes of immune cells was estimated through Cibersort in the training (Figure 5(d)) and whole validation cohorts (Figure 5(e)). Several immune cells were differentially infiltrated between low- and high-risk groups, while three of them showed consistent alterations in both training and whole validation cohorts. To be specific, the amounts of plasma cells in the high-risk group were lower than that in low-risk group, while *M*2-polarized macrophages and regulatory *T* cells (Tregs) were highly infiltrated in high-risk groups.

### 3.6. Antiangiogenic Response Prediction

Based on the “oncoPredict” package, the IC_50_ values of 3 antiangiogenic drugs (cabozantinib, lenalidomide, and cediranib) were calculated for each patient. Results revealed that the IC_50_ values of high-risk group were significantly lower than that in low-risk patients in both training and whole validation cohorts (Figures 5(f) and 5(g)), further indicating that they were more sensitive to antiangiogenic treatments and were suitable for such therapies.

## 4. Discussion

Due to the heterogeneous nature of PCa, satisfactory assessment and management of patients after radical therapy are difficult to accomplish [25, 26]. Therefore, the establishment of effective biomarkers to stratify high-risk patients that easily developed into BCR becomes a critical clinical challenge, thus helping determine whether further therapies are warranted [5]. To address this task, here we identified an angiogenesis-related three-gene signature that precisely and robustly predicts BCR.

Angiogenesis, the process whereby blood vessels develop from a preexisting vascular network, establishes a blood supply to satisfy nutrients and oxygen demands and accomplish other metabolic functions [27]. In growing cancer, the constant production of angiogenesis inducers and correspondingly reduced antiangiogenic factors lead to increased activities of endothelial cells [28]. It has emerged as a hallmark of carcinogenesis and was essential for tumor growth and invasion, leading to metastasis [29, 30]. In this study, by applying ssGSEA, activities of 50 hallmark activities were quantified. Among them, angiogenesis, IFN-*α* response, and EMT were essential risk factors for BCR, while angiogenesis ranks first. PCa patients with high angiogenesis-related scores bear a poor prognosis than low-score patients. Consistently, the same results can be seen in another three independent validation cohorts. As previously reported, indicators of intratumor angiogenic activity, including microvessel density and expression level of VEGF-A, were tightly associated with higher tumor stage and grade, and worse prognosis in PCa [31, 32]. Increased angiogenic activities and higher expression levels of angiogenesis-related genes in BCR in comparison with non-BCR patients revealed that it served as a vital factor in the recurrence and progression of PCa.

In the process of gene signature establishment, besides univariate Cox regression analysis, a random survival forest algorithm was also applied for gene selection [33]; common angiogenesis-related genes selected by both approaches were further put into multivariate Cox regression analysis to develop ARS. In contrast with other clinicopathological factors, higher ARS consistently indicated poor prognosis of PCa patients, and it also has a promising ability in BCR prediction. Previous studies also provided several gene signatures for BCR prediction; however, too many genes included or the lack of multicenter validation limited their application [5]. For example, Abou-Ouf et al. constructed a 10-gene signature for BCR prediction with an AUC of 0.65 [34], while only one cohort was utilized to validate gene signatures in other studies [13, 35]. The AUC values of our signature with only three genes were more than 0.7 in the training cohort and higher than 0.65 in most validation cohorts for BCR. Clinically, BCR indicates a major disease progression and is closely correlated with an increased risk of metastasis for PCa patients [5]. Therefore, we evaluated its performance in metastasis prediction, and results also indicated a higher risk in high ARS patients with an AUC of more than 0.7. The above results were trained and validated in four independent cohorts, which strongly verified the robustness of ARS. Furthermore, it is of great significance to use tumor-risk stratification tools for personalized medicine and thus to choose an optimal management strategy [36], and the ARS allowed us to classify PCa patients after radical therapy into high- and low-risk of early BCR. Given its stable performance and comprehensive validation in multiple cohorts, we trust that this novel angiogenesis-related signature would serve as a potential tool for clinical application.

In the present study, GSEA recognized that the high-risk group divided by ARS was mainly enriched in direct or indirect angiogenesis-related processes, such as “positive regulation of endothelial cell proliferation,” “VEGF receptor signaling pathway,” and “aortic development” [37]. These results further highlighted that more angiogenesis-related activities occurred in the tumors of PCa patients with higher ARS, and it was tightly associated with BCR. As for hallmark activities, EMT and TGF-*β* signaling were significantly enriched in the high-risk group. Several studies have reported that the TGF-*β* signaling pathway contributed to EMT, angiogenesis, migration, and metastases in several malignancies including PCa, while EMT was widely regarded as a certain cancer hallmark [30, 38, 39]. Above results of functional enrichment analyses indicated angiogenesis-related pathways were highly enriched in high-risk patients and further proved the predictive ability from a functional perspective.

The immune microenvironment that includes several subtypes of coexisting immune cells may influence PCa recurrence [40]. In this study, the infiltration of plasma cells was lower in high-risk PCa patients. Similarly, previous studies identified that reduced or nonenriched plasma cell environment independently characterized an aggressive phenotype in localized PCa, which was in agreement with our findings [41]. Additionally, increased infiltration of Tregs and *M*2-polarized macrophages were found in high-risk patients. It has been widely established that Tregs play critical roles in aggravating cancer development and *M*2-polarized macrophages are related to immune suppression and cancer metastasis [42]. In the immunological landscape of PCa, Tregs may contribute to adverse clinical courses as they are associated with advanced diseases [43], while higher numbers of *M*2 macrophages increased the mortality rate [44]. The above three categories of immune cells may contribute to the higher rate of occurrence and poor prognosis of high-risk patients.

Although high levels of angiogenesis-related activities indicated tumor progression in PCa, antiangiogenic therapies failed to provide essential treatment benefits in current clinical trials [10, 11]. One possible explanation is that biomarkers assessing response to antiangiogenic treatment and screening patients who are more likely to take the advantage of antiangiogenic therapies are currently absent [45]. In this research, we evaluated the therapeutic responses to three available angiogenesis inhibitors of each patient based on GDSC database. Our results demonstrated that patients with high ARS or in the high-risk group had lower IC_50_ values in all cohorts, which demonstrated that those patients would be more likely to benefit from antiangiogenic treatments. This novel signature showed potential in identifying specific subgroups of patients who might benefit from antiangiogenic therapies.

Despite promising and robust performance in BCR prediction, there are still some limitations to this study. Firstly, restricted by its retrospective proposal, there are high demands for further prospective studies with larger cohorts to verify the reproducibility and prognostic accuracy of this novel gene signature. Moreover, biological mechanisms and tumor microenvironments occurring in high-risk groups are still unclear and required to be further emphasized in functional research. Although an intriguing phenomenon was observed in drug responses, laboratory and clinical evidence to support this conclusion is worthy of further assessment.

## 5. Conclusion

In summary, we successfully established and validated a novel angiogenesis-related three-gene signature, which could accurately and robustly predict clinical outcomes (BCR and metastasis) and Tregs/*M*2-polarized macrophages infiltration of PCa patients. Moreover, high-risk patients showed better antiangiogenic responses, which may be suitable for such treatments and thus be benefited. Therefore, our gene signature promises to improve prognosis prediction and offer proper management plans for PCa patients after radical therapy.

## Figures and Tables

**Table 1:** Clinical characteristics of PCa patients in four independent cohorts.

Characteristics	TCGA	GSE116918	GSE70770	GSE54460
Total	327	223	201	93

Application	Training	Validation I	Validation II	Validation III

Age (%)				
<60	126 (38.5)	26 (11.7)	43 (21.4)	38 (40.9)
≥60	201 (61.5)	197 (88.3)	68 (33.8)	55 (59.1)
Unknown			90 (44.8)	

Gleason score (%)				
5			2 (1.0)	1 (1.1)
6	17 (5.2)	39 (17.5)	35 (17.4)	10 (10.8)
7	165 (50.5)	88 (39.5)	139 (69.2)	73 (78.5)
8	41 (12.5)	47 (21.1)	12 (6.0)	6 (6.5)
9	101 (30.9)	49 (22.0)	10 (5.0)	3 (3.2)
10	3 (0.9)		1 (0.5)	
Unknown			2 (1)	

pT stage (%)				
*T*1		51 (22.9)		13 (14.0)
*T*2	113 (34.6)	76 (34.1)	80 (39.8)	61 (65.6)
*T*3	208 (63.6)	92 (41.3)	116 (57.7)	11 (11.8)
*T*4	6 (1.8)	4 (1.8)	1 (0.5)	1 (1.1)
Unknown			4 (2.0)	7 (7.5)

Clinical outcomes				
BCR (%)				
No	272 (83.2)	172 (77.1)	139 (69.2)	51 (54.8)
Yes	55 (16.8)	51 (22.9)	62 (30.8)	42 (45.2)
Metastasis (%)				
No		201 (90.1)		
Yes		22 (9.9)		

Follow-up time (months, mean ± SD)	33.94 (26.39)	78.46 (25.64)	55.09 (28.82)	71.40 (38.83)

## Data Availability

The data used to support the findings of this study are available from the corresponding author upon request.

## Supplementary Materials

Supplementary Materials Table S1: univariate Cox analysis of 50 cancer hallmarks for the biochemical recurrence of prostate cancer after radical therapy.
