# Supplementary material for: A Novel Angiogenesis-Related Gene Signature to Predict Biochemical Recurrence of Patients with Prostate Cancer following Radical Therapy
Source: J Oncol. 2022 Jun 28;2022:2448428. doi: 10.1155/2022/2448428 (PMC9256390; doi:10.1155/2022/2448428)
Supplement: Supplementary Materials — Table S1: univariate Cox analysis of 50 cancer hallmarks for the biochemical recurrence of prostate cancer after radical therapy. [file 2448428.f1.docx]

Supplementary Table S1: Univariate Cox analysis of 50 cancer hallmarks for the biochemical recurrence of prostate cancer after radical therapy. HR: hazard ratio.

| ID | HR | HR.95L | HR.95H | *P* value |
| --- | --- | --- | --- | --- |
| Angiogenesis | 1.566237 | 1.209969 | 2.027407 | 0.000656 |
| Mitotic spindle | 1.484081 | 1.095307 | 1.999303 | 0.001366 |
| IFN-α repsonse | 1.350291 | 1.038148 | 1.756285 | 0.02515 |
| G2M checkpoint | 1.349013 | 1.071014 | 1.699171 | 0.011001 |
| Epithelial-mesenchymal transition | 1.322514 | 1.019934 | 1.714859 | 0.034956 |
| E2F targets | 1.30045 | 1.037014 | 1.630806 | 0.022926 |
| TGF-β signaling | 1.277641 | 0.973035 | 1.677601 | 0.077859 |
| Wnt/β-Catenin signaling | 1.274725 | 0.964417 | 1.684877 | 0.088119 |
| IFN-γ response | 1.274299 | 0.980357 | 1.656374 | 0.070034 |
| Allograft rejection | 1.252422 | 0.971897 | 1.613916 | 0.081922 |
| PI3K/AKT/mTOR signaling | 1.224801 | 0.936038 | 1.602645 | 0.139371 |
| Apical junction | 1.211798 | 0.923165 | 1.590675 | 0.166359 |
| Hedgehog signaling | 1.206391 | 0.919614 | 1.582597 | 0.175464 |
| Inflammatory response | 1.197829 | 0.91805 | 1.562872 | 0.183524 |
| IL2/STAT5 signaling | 1.197366 | 0.91811 | 1.561562 | 0.183717 |
| Up-regulated in KRAS signaling | 1.189585 | 0.911956 | 1.551735 | 0.200446 |
| Complement | 1.163153 | 0.890038 | 1.520076 | 0.268364 |
| MYC targets v1 | 1.153132 | 0.887914 | 1.49757 | 0.285307 |
| Coagulation | 1.138054 | 0.874521 | 1.481001 | 0.335912 |
| IL6/JAK/STAT3 signaling | 1.134095 | 0.869441 | 1.479307 | 0.353358 |
| mTORC1 signaling | 1.083298 | 0.828064 | 1.417203 | 0.559444 |
| DNA repair | 1.067659 | 0.824718 | 1.382163 | 0.619191 |
| Down-regulated in UV response | 1.04535 | 0.804967 | 1.357518 | 0.739384 |
| Notch signaling | 1.037765 | 0.799995 | 1.346202 | 0.780089 |
| MYC targets v2 | 1.03483 | 0.79134 | 1.35324 | 0.802481 |
| Hypoxia | 1.025805 | 0.796431 | 1.32124 | 0.84359 |
| Glycolysis | 1.022511 | 0.789198 | 1.324799 | 0.866222 |
| Up-regulated in UV response | 1.005905 | 0.777209 | 1.301895 | 0.964315 |
| Apoptosis | 0.995356 | 0.766375 | 1.292753 | 0.97216 |
| Unfolded protein response | 0.979115 | 0.744247 | 1.288102 | 0.880112 |
| Reactive oxygen species pathway | 0.973824 | 0.749551 | 1.265201 | 0.842566 |
| Heme metabolism | 0.955499 | 0.740121 | 1.233553 | 0.726858 |
| Apical surface | 0.955242 | 0.735911 | 1.239942 | 0.730805 |
| Pancreatic β cells | 0.947942 | 0.728654 | 1.233223 | 0.690426 |
| P53 pathway | 0.892541 | 0.684811 | 1.163282 | 0.400327 |
| Myogenesis | 0.880952 | 0.666361 | 1.164648 | 0.373529 |
| Cholesterol homeostasis | 0.864274 | 0.664898 | 1.123435 | 0.275661 |
| Xenobiotic metabolism | 0.856724 | 0.66693 | 1.10053 | 0.226178 |
| Adipogenesis | 0.850996 | 0.667791 | 1.084462 | 0.192086 |
| Oxidative phosphorylation | 0.840111 | 0.642372 | 1.098717 | 0.20323 |
| Protein secretion | 0.840097 | 0.669728 | 1.053805 | 0.131873 |
| Spermatogenesis | 0.837399 | 0.636671 | 1.101412 | 0.204391 |
| Late estrogen repsponse | 0.830937 | 0.648058 | 1.065424 | 0.144214 |
| Down-regulated in KRAS signaling | 0.823251 | 0.639471 | 1.059848 | 0.131301 |
| Androgen response | 0.814444 | 0.639242 | 1.037665 | 0.096755 |
| Early estrogen repsponse | 0.803508 | 0.622412 | 1.037295 | 0.093162 |
| Peroxisome | 0.797783 | 0.612188 | 1.039645 | 0.094487 |
| Bile acid metabolism | 0.749419 | 0.586057 | 0.958318 | 0.021485 |
| Fatty acid metabolism | 0.712564 | 0.548173 | 0.926252 | 0.011327 |
